# The Transport of Charged Molecules across Three Lipid Membranes Investigated with Second Harmonic Generation

**DOI:** 10.3390/molecules28114330

**Published:** 2023-05-25

**Authors:** Baomei Xu, Jianhui Li, Shuai Zhang, Johar Zeb, Shunli Chen, Qunhui Yuan, Wei Gan

**Affiliations:** 1 Shenzhen Key Laboratory of Flexible Printed Electronics Technology, School of Science, Harbin Institute of Technology (Shenzhen), University Town, Shenzhen 518055, China; 19b958030@stu.hit.edu.cn (B.X.); 20b358012@stu.hit.edu.cn (J.L.); 22b358022@stu.hit.edu.cn (S.Z.); johar@hit.edu.cn (J.Z.); 2 School of Chemistry and Chemical Engineering, Harbin Institute of Technology, Harbin 150001, China; 3 Key Laboratory for Preparation and Application of Ordered Structure Materials of Guangdong Province, College of Chemistry and Chemical Engineering, Shantou University, Shantou 515063, China; chenxl@stu.edu.cn; 4 Shenzhen Key Laboratory of Flexible Printed Electronics Technology, School of Materials Science and Engineering, Harbin Institute of Technology (Shenzhen), University Town, Shenzhen 518055, China; yuanqunhui@hit.edu.cn

**Keywords:** lipid membrane, molecular transport, energy barrier, second harmonic generation, vesicle

## Abstract

Subtle variations in the structure and composition of lipid membranes can have a profound impact on their transport of functional molecules and relevant cell functions. Here, we present a comparison of the permeability of bilayers composed of three lipids: cardiolipin, DOPG (1,2-dioleoyl-sn-glycero-3-phospho-(1′-rac-glycerol), and POPG (1-palmitoyl-2-oleoyl-sn-glycero-3-phospho-(1′-rac-glycerol)). The adsorption and cross-membrane transport of a charged molecule, D289 (4-(4-diethylaminostyry)-1-methyl-pyridinium iodide), on vesicles composed of the three lipids were monitored by second harmonic generation (SHG) scattering from the vesicle surface. It is revealed that structural mismatching between the saturated and unsaturated alkane chains in POPG leads to relatively loose packing structure in the lipid bilayers, thus providing better permeability compared to unsaturated lipid bilayers (DOPG). This mismatching also weakens the efficiency of cholesterol in rigidifying the lipid bilayers. It is also revealed that the bilayer structure is somewhat disturbed by the surface curvature in small unilamellar vesicles (SUVs) composed of POPG and the conical structured cardiolipin. Such subtle information on the relationship between the lipid structure and the molecular transport capability of the bilayers may provide clues for drug development and other medical and biological studies.

## 1. Introduction

The transport of functional molecules into and out of cells is critically important for life [1,2,3]. Passive diffusion is thought to be a fundamental transport mechanism across lipid bilayers. Any external influence or intrinsic change that alters the physicochemical properties of membranes may impact the cellular activities such as nutrient uptake and signal transduction [4,5,6]. It is well known that the composition and structure of lipid membranes crucially determine the membrane permeability for molecules to cross the lipid bilayers [7,8,9,10]. Previous studies have discussed the effects of multiple factors, including the length or/and unsaturation degree of lipid chains, the charge of head groups [11,12,13,14], the addition of other components, etc., on the membrane permeability [15,16,17,18]. At the same time, environmental conditions (e.g., temperature, ionic strength) may distinctly affect the structure of lipid membrane and the transport behaviors of molecules through membranes [19,20,21,22].

The unsaturation degree of lipid chains is recognized to have significant influence on the permeability of membranes. This effect can be easily understood because the saturated lipids with relatively high phase transition temperature are with highly ordered hydrocarbon chains at room temperature (gel state), while unsaturated lipid membranes are in liquid-crystalline state with more fluidity and disordered chains [4,11,23,24]. For example, the water permeability increases with the unsaturation degree of monoglyceride membranes [25]. An approximately five-fold increase in water permeability was observed upon switching diacyl phosphatidylcholine lipids with two saturated C-18 chains to unsaturated diacyl phosphatidylcholine lipids with three double bonds on each chain [26]. By increasing the number of double bonds in mixed unsaturated lipids, the passive permeability for leucine can be increased [13]. B. Ehrenberg et al. found that the permeability of fluorescent markers such as calcein and carboxyfluorescein across lipids increased as the unsaturation degree of the acyl chain increased [27]. It has also been demonstrated that saturated lipid bilayers (e.g., 1,2-dipalmitoyl-sn-glycero-3-phospho-(1′-rac-glycerol)—DPPG, 1,2-distearoyl-sn-glycero-3-phospho-(1′-rac-glycerol)—DSPG) show low or no transport of a hydrophobic ion (malachite green—MG) at room temperature [23,28]. On the contrary, the unsaturated lipid bilayer (e.g., phosphatidylglycerol (PG), phosphatidylserine and phosphatidylcholine (PC)) displays notable transport for probe molecules/ions (e.g., calcein, MG, and D289) [22,23,27,29,30,31].

Therefore, increasing the temperature may change the structure of lipid bilayers and alter their permeability. M.W. Kim et al. reported that although MG had no transport through the saturated DSPG lipids at the temperature of 20 °C, it showed a remarkable transport with the temperature increased above the gel-fluid phase transition temperature of DSPG [28]. A similar temperature effect has been observed for the transport of acetic acid on the lipid membrane of dipalmitoylphosphatidylcholine (DPPC) or for the permeation of a series of indole derivatives across the surface of binary lipid vesicles [32,33].

The length of lipid chains also significantly affects the permeability of molecules [34]. There is a simple understanding that the molecular permeability decreases with the increase in lipid chain length. B. Poolman et al. found that by adding two carbon atoms in lipid chain in the range from 14 to 22 carbons, the permeability of water and weak acids decreased to ca. 67% on average, which was attributed to the inversely proportional relationship between the thickness of the membrane and the permeability coefficient [24]. The permeability coefficient of acetic acid was also observed to decrease with the lipid chain length increase from 12 to 18 carbon [32]. Furthermore, a fluorescent dye, calcein, was also observed to be released much weaker with the increase in alkane chain length through the bilayer of PC lipids [35].

The adsorption and permeation of small molecules on lipid membranes may also be affected by the type of polar head in lipids. For example, A. Guglielmelli et al. found that the interaction of tryptophan enantiomers with single-component lipid membranes can be modulated by the lipid’s head type and its physical state. Tryptophan maintains a more superficial binding through interactions with the hydroxyl glycerol groups in anionic DPPG bilayers, whereas in neutral DPPC, it can penetrate deeper into the membrane [36]. S. Sofou et al. found that the head type in mixed lipid vesicles with asymmetric leaflet compositions had notable effect on daptomycin-induced membrane permeability, which was controlled by the defect distribution of lipid-packing in PG lipid-rich region [12]. However, A. Yamaguchi et al. verified that hemicyanine dyes exhibited essentially the same permeation behaviors in the 1,2-dioleoyl-snglycero-3-phosphate (DOPA) bilayer as that in the DOPG bilayer in HEPES/NaOH aqueous buffer solution, although DOPG with additional hydroxyl groups may have better molecular order in the headgroup region of lipid bilayers [37].

The effect of headgroup charge on the permeability of lipid bilayers is somewhat complicated. K. B. Eisenthal et al. demonstrated that the adsorption of positively charged MG on the surface of the zwitterionic 1-palmitoyl-2-oleoyl-sn-glycero-3-phosphocholine (POPC) membrane was very weak, so the cross-membrane transport was not observable. As negatively charged POPG was introduced with mole fraction from 25% to 100%, the transport rate increased linearly with POPG content and MG concentration, showing a significant effect from electrostatic interaction [38]. A. Yamaguchi et al. also observed that cationic hemi-cyanine dyes could transport through the negatively charged lipid bilayers while zwitterionic hemi-cyanine dyes hardly diffused across the bilayer [37]. L. H. Haber et al. investigated the interaction between charged molecules and lipids influenced by multiple factors including the charge of molecules, the introduction of buffer solution, and salt. It was revealed that positively charged MG had higher adsorption free energy on the lipid surface at low salt environment while more adsorption site was observed at high salt environment [22]. It was also revealed in our group that a faster transport of positively charged D289, MG, and doxorubicin (DOX) across the lipid bilayers on DOPG vesicles may be achieved by introducing salt to create an unbalanced ionic strength on two sides of the lipid bilayers. By weakening the electrostatic attraction on one side while keeping that in the other side unchanged, the cross-membrane transport of charged molecules could be promoted. On the other hand, with the salt introduced to both sides of the lipid bilayers, such a notable promotion was not observed [29]. Therefore, many reports show a notable electrostatic interaction between the lipid membrane and the molecules on it [15,29,30,38,39,40].

It was also reported that the introduction of a membrane rigidifier, cholesterol, can regulate the acyl chain packing and enhance the rigidity of the bilayer [15,41,42,43]. On the other hand, some promoter-like ion carrier can facilitate the transport of molecules. For example, it is found that the transport rate of MG across the DOPG bilayer was notably reduced with 50% cholesterol addition [41]. The permeability of glycine and chlorpromazine is significantly impeded with the addition of cholesterol in lipid bilayers [15,42]. If antibiotic valinomycin or gramicidin A is incorporated in vesicles, the transport of MG can be accelerated [18,44]. It was also revealed in our recent work that the structure of anticancer drug molecules also notably influences their adsorption-embedding and transporting on/through DOPG membranes [45]. The alteration of bilayer composition can also serve as a sensor for detecting and modulating the response and permeability to environmentally relevant small molecules, such as fluoride [46].

**Scheme 1:**
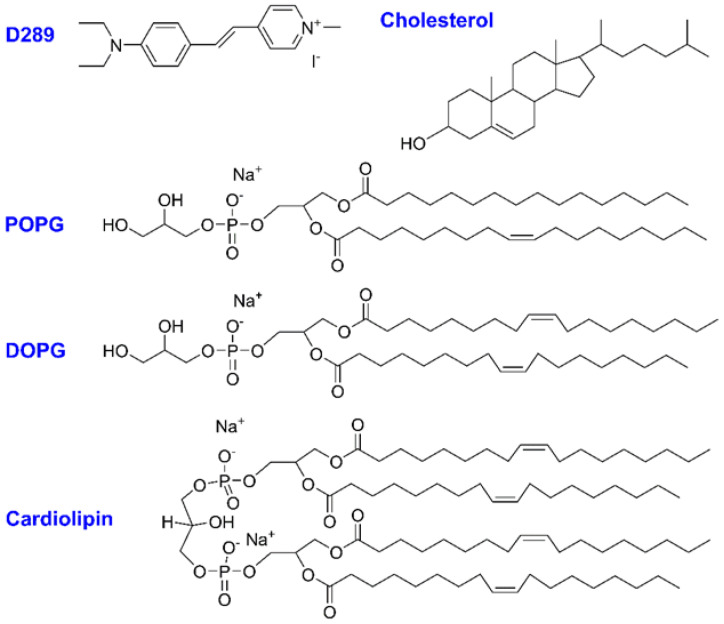
Chemical structures of D289, Cholesterol, and lipids (POPG, DOPG, Cardiolipin).

In this work, we used second harmonic generation (SHG) scattering to study the permeability of a charged dye molecule named D289 across lipid bilayers with various structures (Scheme 1). By monitoring the change in SHG signals scattered from the vesicle surface, the detailed adsorption, embedding, and cross-membrane transport processes of D289 across the lipid bilayers with different structures were revealed. The analysis with the rate of the cross-membrane transport at different temperatures leads to the energy barrier for D289 to penetrate the lipid membranes, thus helping in the understanding of the packing structures and rigidity of the lipid bilayers. Specifically, the introduction of a saturated alkane chain to the DOPG lipid leads to the POPG lipid with elevated saturation degree. The bonding between the head groups of two DOPG molecules by a glycerol bridge makes a more conical cardiolipin molecule. Such structural difference makes notable changes in the permeability of their lipid bilayers. Additionally, the influence of cholesterol addition and the surface curvature of the lipid bilayers are also discussed. The approaches in this work provided an example for analyzing the subtle difference of lipid structures and its influence in the functionality of the lipid membranes.

## 2. Results and Discussion

### 2.1. The Permeability of D289 across Membranes of Different Lipids with GUVs (Giant Unilamellar Vesicles) as a Model

**Figure 1:**
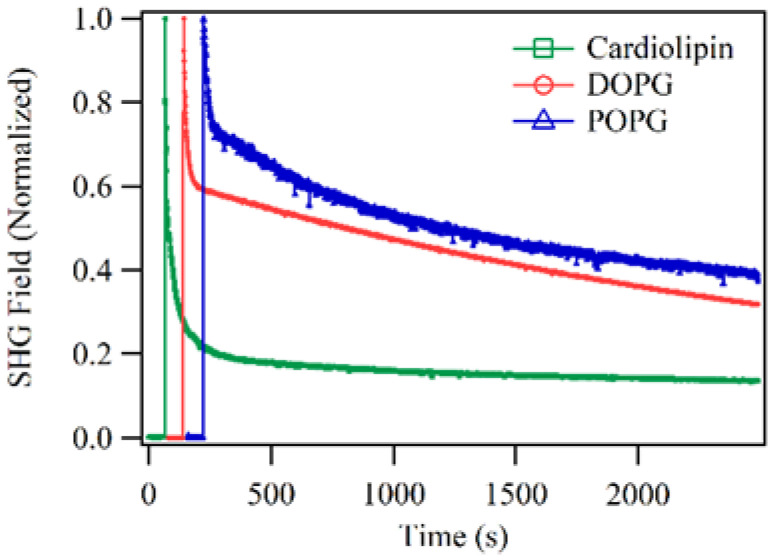
Time-dependent SHG field curves obtained during the interaction of D289 with the GUVs composed of cardiolipin, DOPG, and POPG GUVs. Two of the curves were shifted in *x* direction to make the plot clear.

First, we used GUVs to compare the permeability of the three lipids. GUVs with large size provide relatively flat lipid bilayers, which are similar to the shape of most of the cell surface. On the other hand, SUVs with smaller size provide relatively curved lipid bilayers which are more similar to the surface of some sustained-release capsules. The effect of surface curvature is discussed in latter sections. Figure 1 plots the SHG field, i.e., the square root of the SHG scattering intensities monitored after the addition of D289 in three types of vesicle suspensions. SHG is a second order nonlinear optical phenomenon and is insensitive to molecules in the solution because they are randomly oriented. Once the molecules with relatively large nonlinear efficiency, such as the SHG probe molecules D289 used in this work, adsorb on the interface of lipids, a relatively ordered structure with preferentially oriented molecules is formed, and SHG scattering signal is generated. However, if part of the SHG probe molecules penetrate through the lipids and distribute on another side of the lipid membrane, the SHG signals from two sides of the membrane partly cancel out with each other. Because the SHG field can reflect the density of net-oriented probe molecules on the vesicle surface [4,21,23], these curves can be used to analyze the adsorption and orientation variation of D289 on vesicles, as has been previously illustrated [31,47].

The three curves in Figure 1 show a similar trend which can be interpreted as follows. After the D289 addition, the SHG field first abruptly increases, followed by a rapid and then a slow decline. Based on previous interpretations [19,23,29], the initial increase in the SHG field represents the rapid adsorption of D289 on the surface of vesicles driven mainly by the electrostatic attraction between the negatively charged vesicle surface and the positively charged D289. The adsorption and aggregation of D289 on vesicle surface rapidly create the asymmetric interfacial structures which are the origin of the second order nonlinear polarization of the interfaces [48,49]. This is a very fast dynamic process occurring in seconds. The subsequent fast and slow decrease in the SHG field indicate the flipping-embedding of D289 in the outer surface of vesicles and the transport of D289 across the lipid bilayer from outer to inner surface of the vesicles, respectively [23,28]. This interpretation on the time-dependent SHG curves and the presented physical pictures on the dynamic behavior of D289 is consistent with previous experimental analyses and MD simulations [29,31]. In the previous work [29], S.L. Chen et al. provided a detailed understanding of the cross-membrane transport of D289 on lipid films. It correlated the time-dependent SHG curves with the physical picture of the adsorption, flipping, and cross-membrane transport of D289 on DOPG lipids. In a subsequent work [31], Y. Hou et al. presented the orientation flipping and cross-membrane transport of D289 on DOPG lipids in molecular dynamic (MD) simulation. The attribution of the relatively slow decay in the latter part of the curves to the cross-membrane transport of small charged molecules is also in line with previous reports [28,29,41,47].

**Scheme 2:**
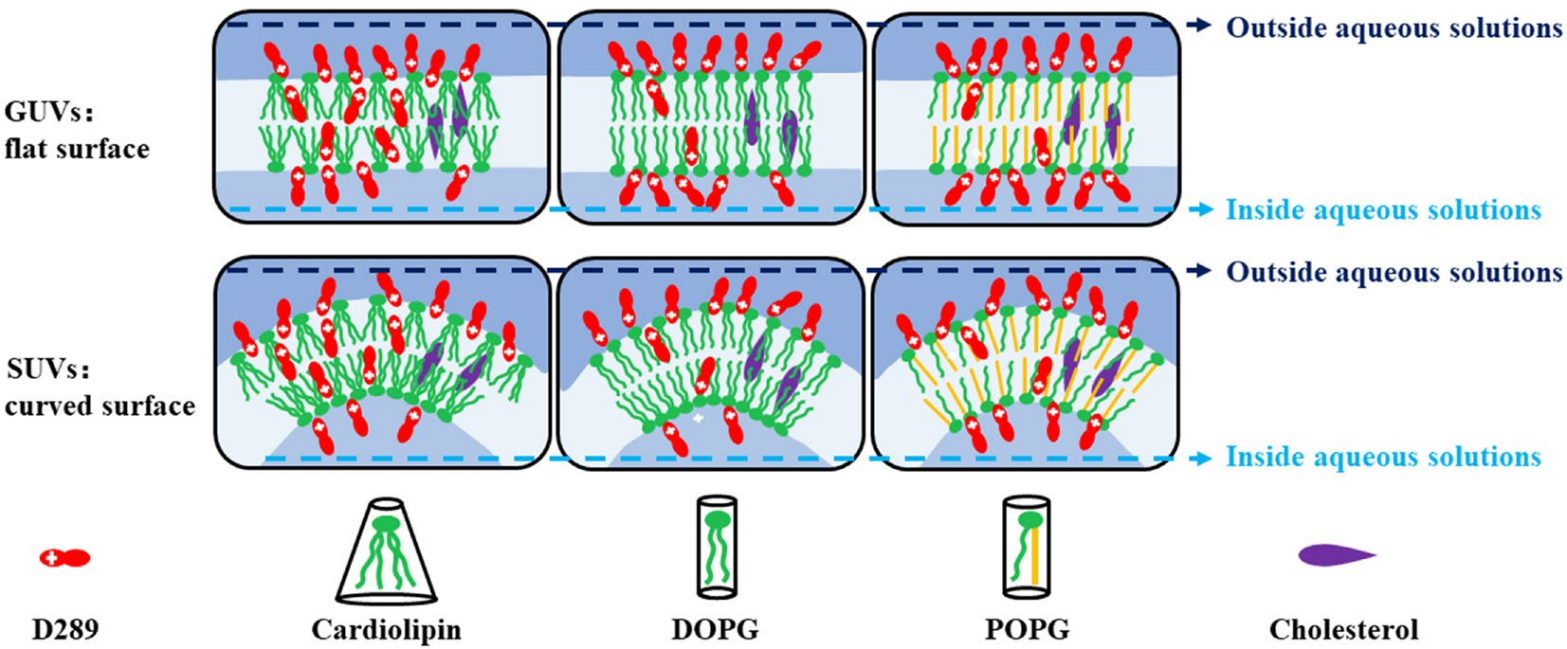
Schematic illustrations showing the interaction between D289/cholesterol and three lipids with flat (GUVs) and curved (SUVs) surface.

It was observed that compared with DOPG and POPG lipids, the SHG curve from cardiolipin presents a more significant rapid decay. It could be related to the structure of cardiolipin which is composed of two phosphatidic acids linked together by a short glycerol bridge [50]. Therefore, it is usually classified as a conical lipid with a relatively small headgroup in comparison to its four fatty acyl chains [51,52]. The conical structure leads to a relatively less compact surface on cardiolipin vesicles, thus allowing more D289 molecules to flip and embed in the lipid membrane, as illustrated by Scheme 2. However, the latter part of the decay is much weaker for the cardiolipin case, indicating that the cross-membrane transport is suppressed. Compared with DOPG lipids with all the alkane chains bearing double bonds, POPG lipids with only half of the alkane chains bearing double bonds, i.e., much higher saturation degree, also show good D289 permeability. That is to say, the introduction of half-saturated alkane chain does not notably alter the rigidity of the lipid bilayers. Otherwise, a relatively slower SHG decay is expected from the POPG experiments. This observation is somewhat contradictory with the generally accepted idea that high saturation degree in the alkane chains tends to prevent the permeability of the lipid bilayers [23,24,26,28].

In order to further evaluate the permeability of different lipid bilayers, we changed the sample temperature during the experiments and measured the energy barrier for the cross-membrane transport of D289 through the lipids. In this work, temperature-dependent experiments were performed with POPG and cardiolipin. The obtained results and the previously reported values for DOPG [19] were used for a comparison.

**Figure 2:**
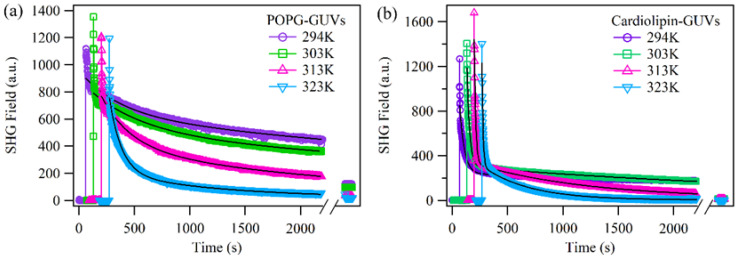
Time-dependent SHG field curves obtained during the interaction of D289 with the GUVs composed of (**a**) POPG and (**b**) cardiolipin under different temperatures as listed in the figure. Three of the curves were shifted in x direction to make the plot clear. The curves on the right side of each frame indicate the remaining SHG field measured after long standing time (beyond 10 h).

As expected, the rate for the cross-membrane transport of D289 accelerated as the temperatures increased (Figure 2). In order to analyze the energy barrier of the D289 transport process, we fitted the decay of the SHG fields with a double exponential function: $E_{2\omega }\left(t\right)=A_{0}+B_{1}exp\left(-\left(t-t_{0}\right)/\tau_{1}\right)+B_{2}exp\left(-\left(t-t_{0}\right)/\tau_{2}\right)$. In order to ensure that the fitting reflects the very slow decay of the SHG field, we recorded the scattered SHG signal with the sample staying overnight and included the data in the fitting. The time constant $\tau_{2}$ corresponding to the slow decay in the latter part of the curves was used to analyze the energy barriers of D289 transport based on the Arrhenius equation, as illustrated in previous reports [19,32,33,53,54].

**Figure 3:**
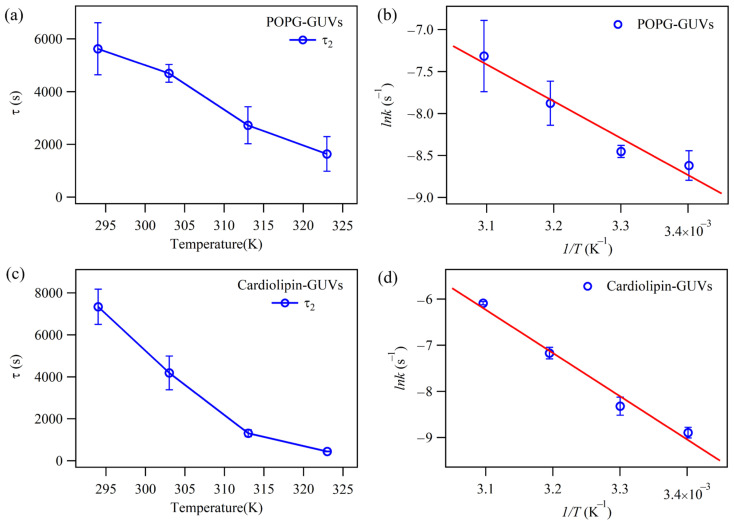
(**a**,**c**) The time constants obtained by exponential fittings of SHG curves in Figure 2. (**b**,**d**) The Arrhenius plot based on the time constants. The solid red lines are from a linear fitting.

As shown in Figure 3a,c, the time constants $\tau_{2}$ obtained by fitting the curves in Figure 2 decrease with the increase in sample temperature. The D289 transport rate *k*, which is reciprocal of the time constant, was used to plot the linear dependent Arrhenius relationship in Figure 3b,d. By this plot, the energy barrier ($E_{a}$) for the transport of D289 across the lipids can by calculated with the Arrhenius equation $lnk=-E_{a}/RT+lnA$ as shown in Table 1.

From Table 1, the energy barrier for D289 to cross the cardiolipin bilayer is slightly higher than that for DOPG bilayer, in line with the observation in Figure 1 showing a slower D289 transport through cardiolipin bilayers. At the same time, the energy barrier for D289 to cross the POPG bilayer is not higher but much lower than that for the DOPG bilayer. This is against the general idea that introducing saturate alkane chains in lipids decreases the permeability of the vesicle [5,23,24,28]. However, in the previous studies, mixed saturated and unsaturated lipids were used to form complex vesicles [33,55]. It is generally accepted that different lipid may form domains with different components in complex vesicles [56,57]. In those studies, the observed permeability of the complex vesicles may present a property between the single component vesicles prepared from the saturated and unsaturated lipids [55,56,58]. Differently, the POPG lipid used in this work is with the saturated and unsaturated alkane chains bonded together, thus preventing the formation of lipid domains bearing different properties. It is known that molecules with long saturated alkane chain provide strong van der Walls interaction between molecules, thus generating a rigid monolayer or bilayer structure [59,60,61,62]. In this scenario, the matching between the structures of the saturated alkane chains plays a crucial role. In our experiment, the saturated alkane chains were held together with the unsaturated ones, preventing the formation of rigid lipids, as illustrated in Scheme 2. Although the 16-carbon chain in POPG is two carbons shorter than the unsaturated chains in DOPG, this difference by itself is not enough to cause the better membrane permeability. The evidence is clearly demonstrated in the DPPG experiments with both 16-carbon chains. Tight arrangement prevented the molecules from transporting through its bilayers. [23]

**Figure 4:**
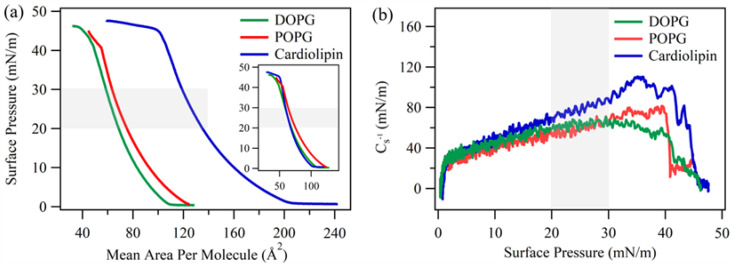
(**a**) Surface pressure (*π*)-area (*A*) isotherms of the monolayers of DOPG, POPG, and cardiolipin. In the inset, the mean area per molecule for cardiolipin (4 chains) is divided by 2 for better comparison with DOPG (2 chains) and POPG (2 chains). (**b**) Compression modulus versus surface pressure based on frame a.

We also measured the surface pressure-area (*π*-*A*) isotherms for POPG, DOPG, and cardiolipin monolayers on deionized water subphase as shown in Figure 4a. According to the literature, the saturated lipid is often considered to have smaller surface area from 40 to 63 Å^2^ in vesicles [63,64,65,66]. The averaged surface area for the head groups of cardiolipin, POPG, and DOPG in vesicles are approximately 130 Å^2^, 66 Å^2^, and 69 Å^2^, respectively [67,68,69]. The corresponding surface pressure is in the range of 20–30 mN/m in Figure 4a, which corresponds to the liquid expansion phase of the monolayers [70,71]. It can be noticed from the highlighted part in Figure 4a that at a certain surface pressure, the mean area occupied per molecule for POPG is slightly larger than DOPG, showing that the mismatching between the saturated and unsaturated alkane chain provided a relatively large “repulsion” between the nearby molecules in the monolayer. This comparison supports that the introduction of saturated alkane chains in POPG does not result in a rigid packing in the lipid bilayers and, thus, cannot prevent the D289 transport.

From these *π*-*A* curves in Figure 4a, the compressional modulus of different lipid monolayers can be calculated by the equation $C_{s}^{-1}=-A\left(d\pi /dA\right)$. This parameter reflects the packing and rigidity of the monolayer under different surface pressures [70]. It is accepted that the higher the value is, the more rigid and ordered the monolayer is. As shown in Figure 4b, the POPG monolayer has a lower compression modulus than the DOPG and cardiolipin monolayers at the surface pressures range of 20–30 mN/m, suggesting the highest resistance to pressure change and confirming the above understanding on the packing order and rigidity of the three lipid monolayers, which can shed light on the permeability of the bi-layer structure in vesicles.

### 2.2. The Effect of Cholesterol on the Permeability of Lipid Bilayers

**Figure 5:**
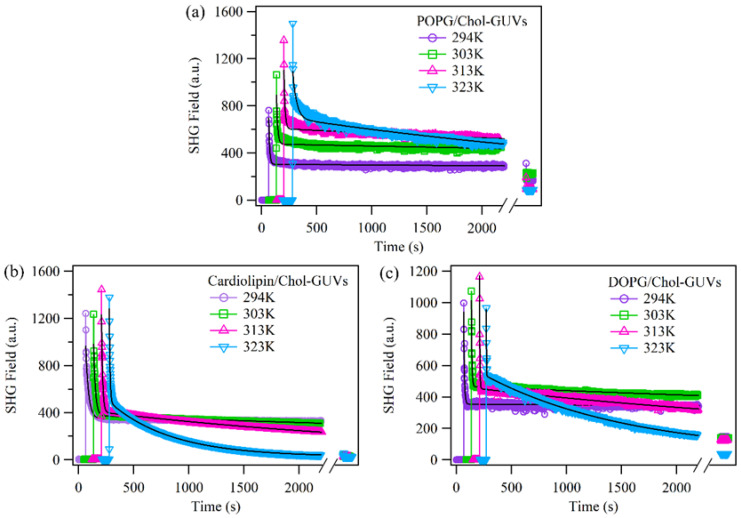
Time-dependent SHG field curves obtained during the interaction of D289 with the GUVs composed of (**a**) POPG/cholesterol, (**b**) cardiolipin/cholesterol, and (**c**) DOPG/cholesterol under different temperature as listed in the figure. Three of the curves were shifted in *x* direction to make the plot clear.

**Figure 6:**
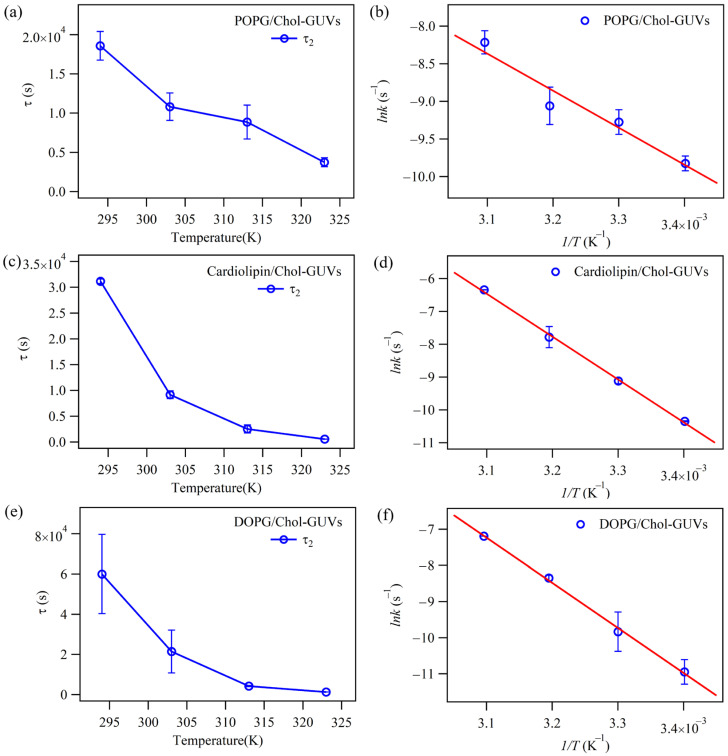
(**a**,**c**,**e**) The time constants obtained by exponential fittings of the SHG curves in Figure 5. (**b**,**d**,**f**) The Arrhenius plot based on the time constants. The solid red lines are from a linear fitting.

It is known that the introduction of cholesterol increases the rigidity and, thus, lowers the permeability of lipid bilayers [16,41]. In this work, 50% (mol ratio) of the lipid was replaced by cholesterol to investigate the effect of cholesterol on the lipid bilayers with three various structures. As shown in Figure 5, after the introduction of cholesterol, GUVs prepared with the three lipids all present less permeability for the D289 transport. The decays of the SHG field, especially at the relatively low temperatures, are very weak. However, based on the recorded SHG signals with the samples stood overnight, part of the D289 molecules eventually transport into the vesicles, resulting in a more symmetric structure for the bilayers on vesicle surface and a notably decreased SHG signal. At the same time, the transport of D289 is also accelerated at increased temperature as is shown in Figure 6a,c,e. With the same analyses as detailed in the Section 2.1, plot the linear dependent Arrhenius relationship as is shown in Figure 6b,d,f, the energy barriers for the D289 transport through various lipid bilayers containing cholesterol were also determined as shown in Table 1.

The results in Table 1 confirmed the effect of cholesterol in rigidifying the lipid bilayers with the relatively higher energy barriers for D289 across the vesicles with cholesterol. However, although the increase in the energy barriers for the transport of D289 on DOPG and cardiolipin vesicles is significant, the increase in that of POPG vesicles is very weak. This observation also reflects that the mismatching between the saturated and unsaturated alkane chains in the POPG bilayers leads to a relatively bad molecular packing, which weakens the influence of cholesterol in rigidifying the lipids.

### 2.3. The Effect of Surface Curvature on the Permeabilities of Different Lipids with SUVs as a Model

**Figure 7:**
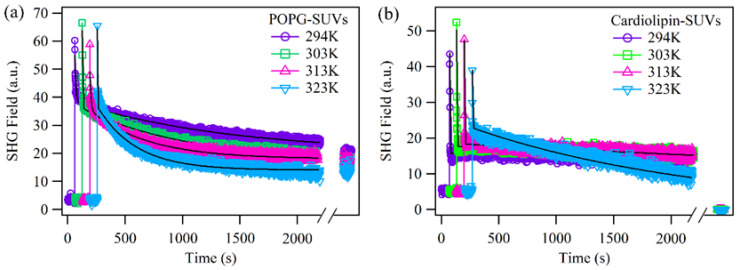
Time-dependent SHG field curves obtained during the interaction of D289 with the SUVs composed of (**a**) POPG and (**b**) cardiolipin under different temperature as listed in the figure. Three of the curves were shifted in *x* direction to make the plot clear.

**Figure 8:**
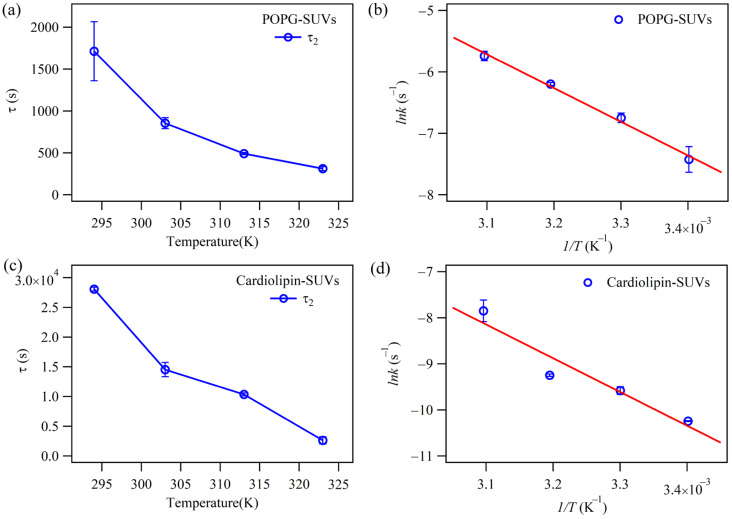
(**a**,**c**) The time constants obtained by exponential fittings of SHG curves in Figure 7. (**b**,**d**) The Arrhenius plot based on the time constants. The solid red lines are from a linear fitting.

It has been recognized that curved lipid bilayers with tension may have different properties compared with the relatively flat bilayers [7,19,52,72]. For a lipid bilayer with the thickness of several nanometers, vesicles as small as tens of nanometers may present this curvature effect [19,52,73]. In our recent work, the permeabilities of D289 across DOPG bilayers on vesicles with different sizes were compared. It was revealed that the energy barrier for the transport of D289 across the DOPG bilayers was notably increased from 68 kJ/mol to 114 kJ/mol with the vesicle diameter changed from ~1000 nm (GUVs) to ~100 nm (SUVs). In this work, a similar comparison was conducted for POPG and cardiolipin with the data obtained from SUVs shown in Figure 7. The energy barriers for D289 transport across the POPG and cardiolipin bilayers in SUVs were also obtained through the Arrhenius plot in Figure 8 and listed in Table 1.

The values in Table 1 provide notably different results for the cardiolipin and POPG bilayers compared with the reported observation from the DOPG vesicles [19]. For the POPG bilayers, the energy barriers of D289 transport through the GUVs surface and SUVs surface are close to each other, indicating that the effect of surface curvature on the rigidity of lipid bilayers is very weak. The mismatching between the saturated and unsaturated chains in POPG may be a possible reason of this observation. For cardiolipin lipid bilayers, the energy barriers for D289 transport through the SUVs surface are slightly smaller than those for GUVs surface. This unexpected result may also come from the fact that cardiolipin has a conical structure. Although conical shaped molecules may benefit from the surface curvature to form a better packing in the inner leaflet of the bilayers, their packing in the outer leaflet of the bilayers can be notably disturbed, as Scheme 2 shows. The energy barrier values for the penetration of D289 through these lipid bilayers, as detected by SHG, are found to be consistent with the activation energy range of 28–128 kJ/mol observed for some organic molecules across vesicles using alternative techniques such as fluorescence spectrophotometry, stopped-flow fluorescence spectrophotometry, molecular sieve chromatography, and a combined method of nuclear magnetic resonance (NMR) line broadening and dynamic light scattering [32,33,74].

**Figure 9:**
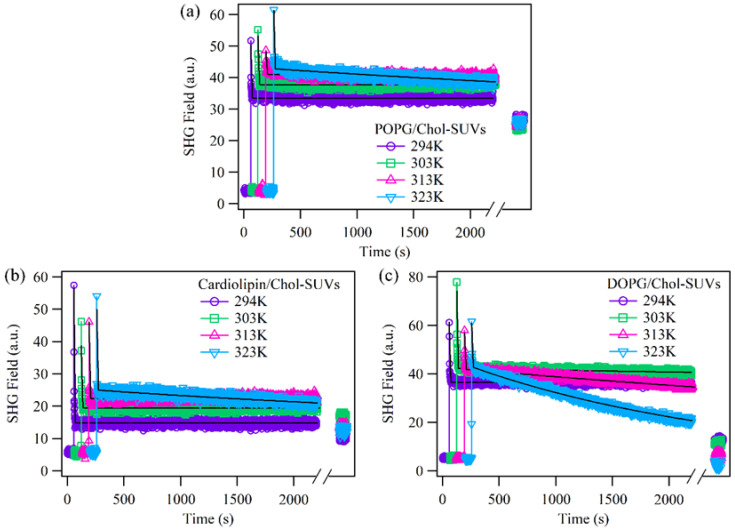
Time-dependent SHG field curves obtained during the interaction of D289 with the SUVs composed of (**a**) POPG/cholesterol, (**b**) cardiolipin/cholesterol, and (**c**) DOPG/cholesterol under different temperature as listed in the figure. Three of the curves were shifted in *x* direction to make the plot clear.

The effect of cholesterol on D289 permeability at the surface of SUVs was also tested with the SHG method. A shown in Figure 9, the cross-membrane transport of D289 is notably suppressed by the cholesterol addition. At relatively low temperature, the latter part of the SHG signal keeps almost flat. For this reason, the fitting of the data and the analysis based on these curves failed to deliver reliable energy barrier values for these experiments. However, the notably suppressed cross-membrane transport observed here is in line with the previously reported observations [15,41,42,43]. Overall, the observations presented in this work show that the influence of the lipid molecular structures, surface curvature, and the introduction of the rigidifier may interact with each other and, thus, lead to tangled and sometimes unexpected results.

## 3. Experimental

### 3.1. Materials

POPG, 99.9%, Cardiolipin, 99.9%, and DOPG, 99.9% were purchased from Avanti Polar Lipids (Alabaster, AL, USA). Cholesterol (99%) and D289 (97%) were purchased from Sigma-Aldrich (St. Louis, MO, USA). Chloroform (AR) was obtained from Tianjin Damao Chemical Reagent Factory (Tianjin, China). Polycarbonate membrane with pore size of 0.1 µm was purchased from GE Healthcare Life Sciences (Piscataway, NJ, USA). Deionized water (18.2 MΩ·cm) was prepared with a water purification system (WP-UP-UV-20) from Sichuan Water Technology Development Co., Ltd. (Chengdu, China).

### 3.2. Vesicles Preparation and Characterization

GUVs and SUVs were produced via a gentle hydration method and the extrusion method, respectively [19,75,76]. Briefly, lipids powder with or without cholesterol at a certain amount was dissolved in chloroform, then a uniformly thin film was formed on a glass bottle by manually rotating the bottle with the chloroform evaporated in a fume hood. The bottle was then placed in vacuum for at least 6 h to remove residual chloroform. The dried film was hydrated by adding deionized water. The above procedures were the same for the generating of both GUVs and SUVs. Then, GUVs was obtained by standing the sealed hydrated lipid solutions for two days at room temperature. To obtain SUVs, the hydrated lipid solutions were subjected to a freeze/thaw (4/45 °C) cycle for 5 times and extruded (reciprocated) for 20 rounds through a 0.1 µm polycarbonate membrane. The final concentration of the produced vesicles was controlled as 1 mM and diluted to be 0.5 mM in SHG experiments.

The diameters of the vesicles were characterized by dynamic light scattering measurements using a Malvern Zetasizer Nano ZS90 with the temperature set as 25 °C. Then, 1 mL of the sample was transferred to the cell with the probing laser emitted at a wavelength of 633 nm. The number mean diameters of the vesicles are listed in Table 2. Each data point shows the mean of three measurements, and the errors represent the standard deviation. It shows approximate diameters as slightly more than 1000 nm for GUVs and close to 100 nm for SUVs irrespective of lipid species and the presence/absence of cholesterol. The polydispersity index for the GUVs is approximately 0.3, whereas for SUVs, it is approximately 0.1. The diameters of the vesicles were also checked after the experiments to ensure that the vesicles were stable.

### 3.3. Second Harmonic Generation (SHG) Measurements

**Scheme 3:**
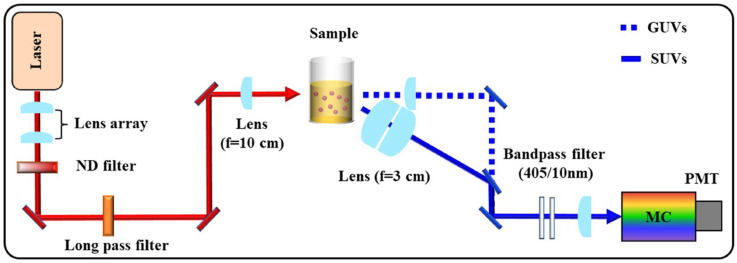
The setup for the SHG experiments.

The SHG experimental setup (Scheme 3) is similar to that descried in a previous report [19]. A Ti: sapphire oscillator (MaiTai-HP, Spectra-Physics, Santa Clara, CA, USA) generated horizontally polarized laser with ~100 fs pulses at the wavelength of 810 nm and repetition rate of 80 MHz. The power was adjusted to 300 mW for the experiments. The laser passed through a long pass filter (FGL9, >720 nm, Thorlabs, Newton, NJ, USA) to block the SH signals generated before the sample and was focused on the sample by a lens with a focal length of 10 cm. The sample was placed in a cylindrical quartz cell with an inner diameter of 13 mm and stirred during the test to avoid the heating effect.

The scattered SHG signal was collected in the forward direction by a plano-convex lens (Ø = 2.54 cm, f = 10 cm, Thorlabs) in experiments with GUVs. For experiments with SUVs, the signal was collected at 35° relative to the incident light direction by a pair of lenses (Ø = 5.08 cm, f = 3 cm, Thorlabs). These collecting directions and areas ensure that the majority of the SHG scattering from the vesicle surface can be probed for both GUVs and SUVs at their respective scattering directions as previously reported [19,31]. The collected signals were filtered by two bandpass filters (FGB37S, 335–610 nm, Thorlabs) to remove the residual laser and analyzed by a monochromator (omni1509, Zolix Instruments Co., Ltd., Beijing, China), and then they were collected with a photomultiplier tube (PMT, Hamamatsu R-1527p, Hamamatsu, Japan). The voltage of the PMT was set as −900 V for the SUVs experiments and −750 V for the GUVs experiments. A preamplifier (SR445A, Stanford Research System, Sunnyvale, CA, USA) was used to amplify the signal by a factor of 5. The amplified signals were then analyzed by a single photon counter (SR400, Stanford Research System) with the data recorded by a computer. The temperature and humidity of the laboratory were kept at 21 ± 1 °C and 40 ± 5%, respectively. In the experiment, the temperature of sample was controlled by a heater (Songdao Heating Sensor Co., Ltd., Shanghai, China) surrounding the sample cell.

Because the emitted SHG signals were partially absorbed/scattered by the vesicles and D289 molecules in the samples, all obtained intensities were corrected based on the extinction spectra of the samples. The hyper-Rayleigh scattering from D289 solution was then subtracted from the corrected signal to acquire the SHG scattering from the vesicles surface.

### 3.4. Surface Pressure-Area Isotherm of Lipid Monolayers

The surface pressure-area (*π*-*A*) isotherms were performed with a KSV Langmuir trough (KN1003, KSV Instruments, Ltd., Helsinki, Finland). The Teflon trough was equipped with two symmetric barriers and controlled using KSV Nima software (Version 2.4) with the surface pressure measured with a Wilhelmy plate during compression. The trough and two barriers were rinsed several times with ethanol and deionized water before the measurements. For preparing a Langmuir monolayer, an appropriate amount of the chloroform solution of lipids was spread on the water surface with a Hamilton microsyringe in a dropwise manner. The lipid films were compressed at a constant rate of 8 mm/min after waiting for about 15 min to allow the chloroform to evaporate.

## 4. Conclusions

The transport of small, charged molecules through lipid membranes is crucial for life, so it has been intensively investigated. Multiple factors may affect the passive transport of molecules through lipids. In this work, the transport of D289 through the bilayers of three lipids (DOPG, POPG, and cardiolipin) influenced by the temperature, the chemical structure of lipids, the curvature of the membrane, and the introduction of cholesterol in the lipids were investigated. Based on the SHG analysis, the introduction of saturated alkane chains in the lipids does not inevitably increase the rigidity of the lipid membrane. The mismatching between the saturated chains and the unsaturated chains may lead to a loosely packed lipid structure, thus benefiting the molecular transport. Specifically, POPG bilayers with half of the alkane chain saturated do not present lower permeability compared with the DOPG bilayers with all the alkane chains having double bonds. Because of the relatively loose packing structures, the introduction of cholesterol or surface curvature has less profound effect on increasing the rigidity of lipid membrane than that for DOPG membrane.

As to the comparison between the DOPG and the cardiolipin vesicles, their similar alkane structures lead to similar D289 permeability for the relatively flat bilayer. The introduction of cholesterol also caused similar influence on their rigidity. However, in SUVs with relatively high surface curvature, the more conical structure of cardiolipin results in a relatively worse packing structure in the outer leaflet of the bilayers on the surface of SUVs, thus providing a relatively low energy barrier for D289 transport.

In this work, the permeability of the lipid bilayers and the corresponding structural information are mainly revealed by the SHG method that monitors the orientational flipping of D289 on the surface of vesicles. More specific packing structures of the lipid bilayers are expected to be revealed with another interface specific nonlinear optical spectroscopic method, Sum Frequency Generation-Vibrational Spectroscopy (SFG-VS). Theoretical simulation is also expected to be able to provide some structural and dynamic information on the interaction between small molecules and lipid bilayers. Such information is still highly desired for the understanding of the behavior of drugs and other functional molecules in the lipid membranes. This work demonstrates that by comparing the energy barriers for small molecules to penetrate different lipid membranes, detailed information on the packing structure and rigidity of lipid bilayers may be revealed. All these studies are highly valuable for the drug and medical studies with the interaction between small molecules and lipid membranes involved.

## Figures and Tables

**Table 1:** The energy barrier ($E_{a}$) of D289 transport through different lipid bilayers from Figure 3 (POPG and cardiolipin, data from two experiments were used to obtain the mean and standard deviation) and previous reports (DOPG) [19].

Lipids	$E_{a}$ (kJ/mol) GUVs—Non Chol	$E_{a}$ (kJ/mol) GUVs—Chol	$E_{a}$ (kJ/mol) SUVs—Non Chol
POPG	36.5 ± 7	40.9 ± 2	45.6 ± 7
cardiolipin	77.9 ± 3	108.6 ± 2	60.9 ± 6
DOPG	68 ^a^	103.8 ± 12	116 ^a^

^a^ From refs.

**Table 2:** Number mean diameters (nm) of the vesicles.

Lipids	SUVs		GUVs	
	Non-Chol	With-Chol	Non-Chol	With-Chol
POPG	94 ± 3	99 ± 3	1320 ± 150	1380 ± 280
cardiolipin	89 ± 2	97 ± 2	1450 ± 180	1450 ± 170
DOPG	97 ± 3	100 ± 1	1230 ± 170	1290 ± 190

## Data Availability

The data presented in this study are available within the article.
